# Supplementary material for: The RNA Helicase Ded1 from Yeast Is Associated with the Signal Recognition Particle and Is Regulated by SRP21
Source: Molecules. 2024 Jun 20;29(12):2944. doi: 10.3390/molecules29122944 (PMC11206880; doi:10.3390/molecules29122944)
Supplement: Supplementary file 1 [file molecules-29-02944-s001.zip › molecules-3047294-supplementary.pdf]

The DEAD-box RNA helicase Ded1 from yeast is associated with the signal recognition particle and is regulated by SRP21.

Hilal Yeter-Alat, Naïma Belgareh-Touzé, Agnès Le Saux, Emmeline Huvelle, Molka Mokdadi, Josette Banroques, and N. Kyle Tanner*

Supplementary Table S1. Sucrose gradients fractions; nano-LC ESI MS/MS analysis[a]

Protein[b]	Fraction(s)[c]	Meta Score[d]	#Spectres[e]	SC%[f]	RMS (ppm)[g]
Ded1	6	213.4	7	14.2	6.60
Ded1	7	1455.7	50	50.8	8.04
SRP14	7	80.7	4	13.0	5.25
SRP21	7	255.5	10	28.7	7.30
SRP54	7	229.4	6	8.9	6.40
SRP68	7	88.2	2	2.2	8.01
ENO2	6	5771.5	639	96.6	5.68
ENO2	7	4741.5	359	94.7	6.50
SSA2	6	4335.6	280	84.4	10.24
SSA2	7	3999.2	226	74.5	8.37
FBA1	6	2125.7	186	80.5	6.52
FBA1	7	1653.2	116	79.7	6.32

[a] Data was collected and analyzed as previously described [1]. In brief, 0.5 ml fractions were collected starting from the top of the gradient and subjected to nano-liquid-chromatography electron-spray mass spectrometry analysis

[b] ENO2, SSA2 and FBA1 are reference proteins; they showed the strongest signals in the fraction at the top of gradient.

[c] Fractions correspond to those shown in Supplemental Figure S7 of Senissar *et al.* [1].

[d] Mascot probably-based scoring.

[e] Spectral counting; the same peptide is fragmented up to six times over a mean elution time of 30 seconds.

[f] Percentage of the protein sequence covered.

[g] Mean error in ppm.

Supplementary Table S2. Ded1-IgG Pull-down of sucrose gradients fractions; Nano-LC ESI MS/MS analysis[a]

Protein[b]	Fraction(s)[c]	Meta Score[d]	#Peptides[e]	SC%[f]	RMS (ppm)[g]
Ded1	6	413.4	25	40.6	3.51
Ded1	7	368.2	8	15.6	1.73
SRP14	6	387.0	9	51.4	2.54
SRP14	7	88.2	2	13.0	2.32
SRP21	6	385.5	6	50.3	3.15
SRP21	7	95.4	2	13.2	1.99
Sec65	6	345.1	7	31.9	2.69
SRP68	6	66.4	1	2.2	0.94
ENO2	6, 7	—	—	—	—
SSA2	6	395.9	9	21.3	2.83
SSA2	7	—	—	—	—
FBA1	6, 7	—	—	—	—

[a] Data was collected and analyzed as previously described [1]. Equivalent fractions as shown in Table 1 were subjected to IgG-Ded1 pull-downs with protein-A Sepharose beads and subjected to mass spectrometry analysis.

[b] ENO2, SSA2 and FBA1 are reference proteins; they showed the strongest signals in Table 1.

[c] Fractions correspond to those shown in Supplemental Figure S7 of Senissar *et al.* [1].

[d] Mascot probably-based scoring.

[e] Number of peptide fragments recovered.

[f] Percentage of the protein sequence covered.

[g] Mean error in ppm.

Supplementary Table S3. Protein characteristics[a]

Protein	Gene	Length (aa)	MW (gm/mole)	pK_i	Abundance[b]	Half life (hr)[c]
Ded1	YOR204W	604	65554.7	7.98	25034 ± 5043	9.1
SRP14	YDL092W	146	16442.1	10.38	5858 ± 1306	9.6
SRP21	YKL122C	167	18451.7	11.14	4248 ± 1211	7.9
SRP54	YPR088C	541	59630.3	9.04	8411 ± 2308	7.0
Sec65	YML105C	273	31177.3	9.62	5632 ± 2232	9.7
SRP68	YPL243W	599	69014.7	9.15	8214 ± 1962	9.9
SRP72	YPL210C	640	73568.8	10.0	6363 ± 1828	9.4
SRP101	YDR292C	621	69276.8	7.19	4444 ± 2444	9.9
SRP102	YKL154W	244	26975.3	8.02	5274 ± 1935	10.1

[a] Data taken from https://www.yeastgenome.org.
[b] Median abundance (molecules/cell).
[c] Data from [2].

Supplementary Table S4. Oligonucleotides used in this study

**Name**	**Name oligo**	**5'—3' sequence[a]**
SCR1 RNA	SCR1_up2	GCC TAT **GGA TCC** TAA TAC GAC TCA CTA TAG GGC TGT AAT GGC TTT CTG GT
	SCR1_low	GCC TAT **CTC GAG TTT AAA** AAT ATG GTT CAG GAC ACA CT
SCR1ΔAlu RNA	SCR1dAlu_up	GCC TAT **GGA TCC** TAA TAC GAC TCA CTA TAG GGT CGT AAA TTT GTC CTG GGC A
	SCR1dAlu_low	GCC TAT **AAG CTT TTT AAA** ACC GCC AAA TTA AAC CGC
SCR1ΔS1 RNA	pUC18_5'	CCG TAT TAC CGC CTT TGA GTG
	SCR1dS1_low	CGC CTC CAT CAC GGG CGAA CCC GCA AAG ATC GAT TTA TTA TAG C
	pUC18_3'	GGT GTG AAA TAC CGC ACA GA
	SCR1dS1_up	ATC GAT CTT TGC GGG TTCG CCC GTG ATG GAG GCG G
SCR1 (northern)	SCR1_Northern	ATA AAA CTC CCC TAA CAG CGG TGA
PGK1 (northern)	PGK1_357	TCT TCG ATG TGG TAA CGC AAG TTT
RPL20B (northern)	RPL20B_Northern	CAG TAA CGA GAC TTG GCG ATG AC
SCR1 (RT-PCR)	SCR1_fwd	CAA ATC CTT CCT CGC GGC TA
	SCR1_rev	CGC CAA ATT AAA CCG CCG AA
PGK1 (RT-PCR)	PGK1_fwd	TTG GAA AAC TTG CGT TAC CAC
	PGK1_rev	CTG GCA AGA CGA CTT CGA CA
RPL20B (RT-PCR)	RPL20B_fwd	TTA CCA ACT GAA TCC GTT CCA
	RPL20B_	GGT CTC TTG TAA GAG AAA GTC T
SRP14	SRP14_up	GCC TAT **ACT AGT CAT ATG** GCA AAT ACT GGC TGT TTA TCA
	SRP14_low	GCC TAT **CTC GAG** GTT TTT CTT CGC TAC CTT GT
SRP21	SRP21_up	GCC TAT **ACT AGT CAT ATG** TCT GTG AAA CCC ATT GA
	SRP21_low	GCC TAT **CTC GAG** ACG CTT TTT TTT GCC CTT GT
SEC65	Sec65_up	GCC TAT **ACT AGT CAT ATG** CCT AGA TTA GAA GAG ATT GA
	Sec65_low	GCC TAT **CTC GAG** TCT TCT AAC TAC TTT GTA CTT ATT TTT TGG T
SEC65 (pET19b)	Sec65-pET_up	GCC TAT **CTC GAG** ATG CCT AGA TTA GAA GAG ATT GAC GA
	Sec65-pET_low	GCC TAT **GGA TCC** TCA TCT TCT AAC TAC TTT GTA CTT AT
SRP54	SRP54_up	GCC TAT **ACT AGT CAT ATG** GTT TTG GCT GAT TTG GGG A
	SRP74_low	GCC TAT **CTC GAG** GCC CAT ACC GAA TTG TTT TGC CA

SRP68	SRP68_up	GCC TAT **ACT AGT CAT** <u>**ATG** GTT GCC TAT TCT CCA ATC</u>
	SRP68_low	GCC TAT **CTC GAG** <u>ACG ACC AAA TAG GCC CA</u>
SRP68 (pET19b)	SRP68_up2	GCC TAT **CTC GAG** <u>ATG GTT GCC TAT TCT CCA ATC</u>
	SRP68_low2	GCC TAT **GGA TCC** <u>ACG ACC AAA TAG GCC CA</u>
SRP72	SRP72_up	GCC TAT **ACT AGT CAT** <u>**ATG** GCT AAA GAT AAT TTA ACT AAT TTG C</u>
	SRP72_low	GCC TAT **CTC GAG** <u>TTT ACG GCC CTT CTT CTT GT</u>
SRP101	SRP101_up	GCC TAT **GGA TCC CAT** <u>**ATG** TTC GAC CAA TTA GCA GTC T</u>
	SRP101_low	GCC TAT **CTC GAG** <u>AGA CAT TAA TGT ATT AAC AGC CCA</u>
SRP102	SRP102_up	GCC TAT **GGA TCC CAT** <u>**ATG** CTT AGT AAT ACA CTT ATT ATT GCC T</u>
	SRP102_low	GCC TAT **CTC GAG** CAG TTT TTC ATC TAT CCA TTC GC
SRP21 Δ73Cter	SRP21_low	GCC TAT **CTC GAG** <u>TTA ACG CTT TTT TTT GCC CT</u>
	SRP21d273_low	GCC TAT **CTC GAG** <u>TTA GTT ATT TTT CTT CTT CGA CTG TGC</u>
GFP & mCherry	GFP/mCherry+XhoI-ed	GCC TAT **CTC GAG** <u>GGA GCA GGT GCT GGT</u>
	GFP/mCherry_SalI-ed	GCC TAT **GTC GAC TTA** <u>**CTT** GTA CAG CTC GTC CA</u>

[a]The oligonucleotides that were used are as shown, where the regions of complementarity are underlined and restriction sites are shown in bold.

Supplementary Table S5. Constructs used in this study

Name	Description	Source or reference
pMW295	*SRP21-SRP71-SEC65 (URA3/2µ)*	[3]
pMW299	*SCR1-SRP54-SRP68-SRP14 (LEU2/2µ)*	[3]
*2HA*_p424	*2HA, ADH/CYC1 (TRP1/2µ)*	[4]
*2HA-SRP14*_p424	*2HA-SRP14,ADH/CYC1 (TRP1/2µ)*	This study
*2HA -SRP21*_p424	*2HA-SRP21,ADH/CYC1 (TRP1/2µ)*	This study
*2HA-SEC65*_p424	*2HA-SEC65,ADH/CYC1 (TRP1/2µ)*	This study
*2HA-SRP54*_p424	*2HA-SRP54,ADH/CYC1 (TRP1/2µ)*	This study
*2HA-SRP68*_p424	*2HA-SRP68,ADH/CYC1 (TRP1/2µ)*	This study
*2HA-SRP72*_p424	*2HA-SRP72,ADH/CYC1 (TRP1/2µ)*	This study
*2HA-SRP101*_p424	*2HA-SRP101,ADH/CYC1 (TRP1/2µ)*	This study
*2HA-SRP102*_p424	*2HA-SRP102,ADH/CYC1 (TRP1/2µ)*	This study
p413	*ADH/CYC1 (HIS3/CEN)*	ATCC (#87370)
*2HA-SRP21Δ73Cter*_p413	*2HA-SRP21Δ73Cter,ADH/CYC1 (HIS3/CEN)*	This study
*SRP14-GFP*_p413	*SRP14-GFP, ADH/CYC1 (HIS3/CEN)*	This study
*SRP21-GFP*_p413	*SRP21-GFP, ADH/CYC1 (HIS3/CEN)*	This study
pET22b	*6HIS (AMP)*	Novagen (#69744-3)
*SRP14*_pET22b	*SRP14-6HIS (AMP)*	This study
*SRP21*_pET22b	*SRP21-6HIS (AMP)*	This study
*SRP72*_pET22b	*SRP72-6HIS (AMP)*	This study
*SRP54*_pET22b	*SRP54-6HIS (AMP)*	This study
*SRP101*_pET22b	*SRP101-6HIS (AMP)*	This study
*SRP102*_pET22b	*SRP102-6HIS (AMP)*	This study
*SRP21Δ73Cter*_pET22b	*SRP21Δ73Cter-6HIS (AMP)*	This study
*DED1*_pET22b	*DED1-6HIS (AMP)*	[5]
*DED1-GAT*_pET22b	*ded1-GAT-6HIS (AMP)*	[6]

pET19b	*6HIS (AMP)*	Novagen (#69677-3)
*SEC65*_pET19b	*6HIS-SEC65 (AMP)*	This study
*SRP68*_pET19b	*6HIS-SRP68 (AMP)*	This study
p415	*ADH/CYC1 (LEU2/CEN)*	ATCC (#87374)
pYM27-*EGFP-KanMX4*	*EGFP (KanMX4, AMP)*	Euroscarf [7]
*GFP*_p415	*GFP, ADH/CYC1 (LEU2/CEN)*	This study
*ded1-DQAD-GFP*_p415	*ded1-DQAD-GFP, ADH/CYC1 (LEU2/CEN)*	This study
GPD-p415	*GPD/CYC1 (LEU2/CEN)*	ATCC (87358)
*GPD-DED1*_p415	*GPD-DED1, GPD/CYC1 (LEU2/CEN)*	This study
*GPD-ded1-F162C*_p415	*GPD-ded1-F162C, GPD/CYC1 (LEU2/CEN)*	This study
HA-ded1ΔC_p415	*HA-ded1ΔC, ADH/CYC1 (LEU2/CEN)*	[8]
*DED1*_YCplac111	*DED1 (LEU2/CEN)*	[9]
p416	*ADH/CYC1 (URA3/CEN)*	ATCC (#87376)
pFA6a-*mCherry-NatNT2* (PFM699)	*mCherry, (NatNT2, AMP)*	Addgene (74636)[10]
*MCHERRY*_p416	*mCherry, ADH/CYC1 (URA3/CEN)*	This study
ded1-DQAD-mCh p416	*DED1-DQAD-mCherry, ADH/CYC1 (URA3/CEN)*	This study
*KAR2-RFP*_YIplac204	*TKC-DsRedExpress2-HDEL, TPI/CYC1 (TRP1/Int)*	Addgene (#21770)
p414	*ADH/CYC1 (TRP1/CEN)*	ATCC (#87372)
*DED1*_p414	*DED1, ADH/CYC1 (TRP1/CEN)*	This study
*ded1-F162C*_p414	*ded1-F162C, ADH/CYC1 (TRP1/CEN)*	This study
*T7-SCR1*_pUC18	*SCR1 (AMP)*	This study
*T7-SCR1ΔAlu*_pUC18	*SCR1ΔAlu (AMP)*	This study
*T7-SCR1ΔS1*_pUC18	*SCR1ΔS1 (AMP)*	This study
*T7-Actin*_BS	*preACTIN (AMP)*	[11]

Supplementary Table S6. Yeast and bacterial strains used in this study

Name	Strain	Genotype	Source or reference
		Saccharomyces cerevisiae	
BY4742	BY4742	*MATα his3Δ1 leu2Δ0 ura3Δ0 lys2Δ1*	Euroscarf
G50	W303 corrected	*MATα ura3-1 trp1-1 leu2-3,112 his3-11,15 can1-100 RAD5 ADE2*	Gift from M. Lisby and R. Rothstein
TET-SRP21	TH_4909	*pSRP21::kanR-tet07-TATA URA3::CMV-tTA MATa his3-1 leu2-0 met15-0*	Dharmacon
TET-SRP14	TH_4306	*pSRP14::kanR-tet07-TATA URA3::CMV-tTA MATa his3-1 leu2-0 met15-0*	Dharmacon
TET-SEC65	TH_6981	*pSEC65::kanR-tet07-TATA URA3::CMV-tTA MATa his3-1 leu2-0 met15-0*	Dharmacon
TET-SRP68	TH_3180	*pSRP68::kanR-tet07-TATA URA3::CMV-tTA MATa his3-1 leu2-0 met15-0*	Dharmacon
TET-SRP72	TH_3294	*pSRP72::kanR-tet07-TATA URA3::CMV-tTA MATa his3-1 leu2-0 met15-0*	Dharmacon
TET-SRP101	TH_7193	*pSRP101::kanR-tet07-TATA URA3::CMV-tTA MATa his3-1 leu2-0 met15-0*	Dharmacon
GFP-SRP14	BY4741	*MATa his3Δ1 leu2Δ0 met15Δ0 ura3Δ0 SRP14-GFP::HIS3MX6*	Life Technologies
GFP-SRP21	BY4741	*MATa his3Δ1 leu2Δ0 met15Δ0 ura3Δ0 SRP21-GFP::HIS3MX7*	Life Technologies
xpo1-T539C	MNY8	*MATa, ΔCRM1::KANr leu2− his3− trp1− ura3− < pDC-XPO1T539C-LEU2/CEN >*	[12]
ded1Δ	W303	*MATα ura3-1 ade2-1 leu2-3,112 trp1-1*	[5]

		DED1/ded1::HIS3MX6 <Ded1/yCPlac33-ura3>	
sec61-ts	RDM 15-5B	*MATα sec61-2, pep4-3, ura3-52, leu2-3,-112, ade2-1*	[13]
sec62-ts	RDM 50-94C	*MATα sec62-1, ura3-52, leu2-3,112, his4, suc+/-*	[13]
KAR2-RFP in sec 61-ts	*sec61-ts*	*MATα sec61-2, pep4-3, ura3-52, leu2-3,-112, ade2-1 trp1-1::DsRedExpress2-HDEL-TRP*	This study
KAR2-RFP in sec 62-ts	*sec62-ts*	*MATα sec62-1, ura3-52, leu2-3,112, his4, suc+/- trp1-1::DsRedExpress2-HDEL-TRP*	This study
G49	W303 corrected	*MATa ura3-1 trp1-1 leu2-3,112 his3-11,15 can1-100 RAD5 ADE2*	Gift from M. Lisby and R. Rothstein
KAR2-RFP in G49	W303 corrected (G49)	*MATa ura3-1trp1-1 leu2-3,112 his3-11,15 can1-100 RAD5 ADE2 trp1-1::DsRedExpress2-HDEL-TRP*	This study
		Escherichia coli	
Rosetta (DE3)	70954	*F- ompT hsdSB(rB- mB-) gal dcm*	Novagen
DH5α	DH5α	*F^- φ80lacZΔM15 Δ(lacZYA-argF)U169 recA1 endA1 hsdR17(r_K^-, m_K^+) phoA supE44 $λ^-$ thi-1 gyrA96 relA1*	New England Biolabs

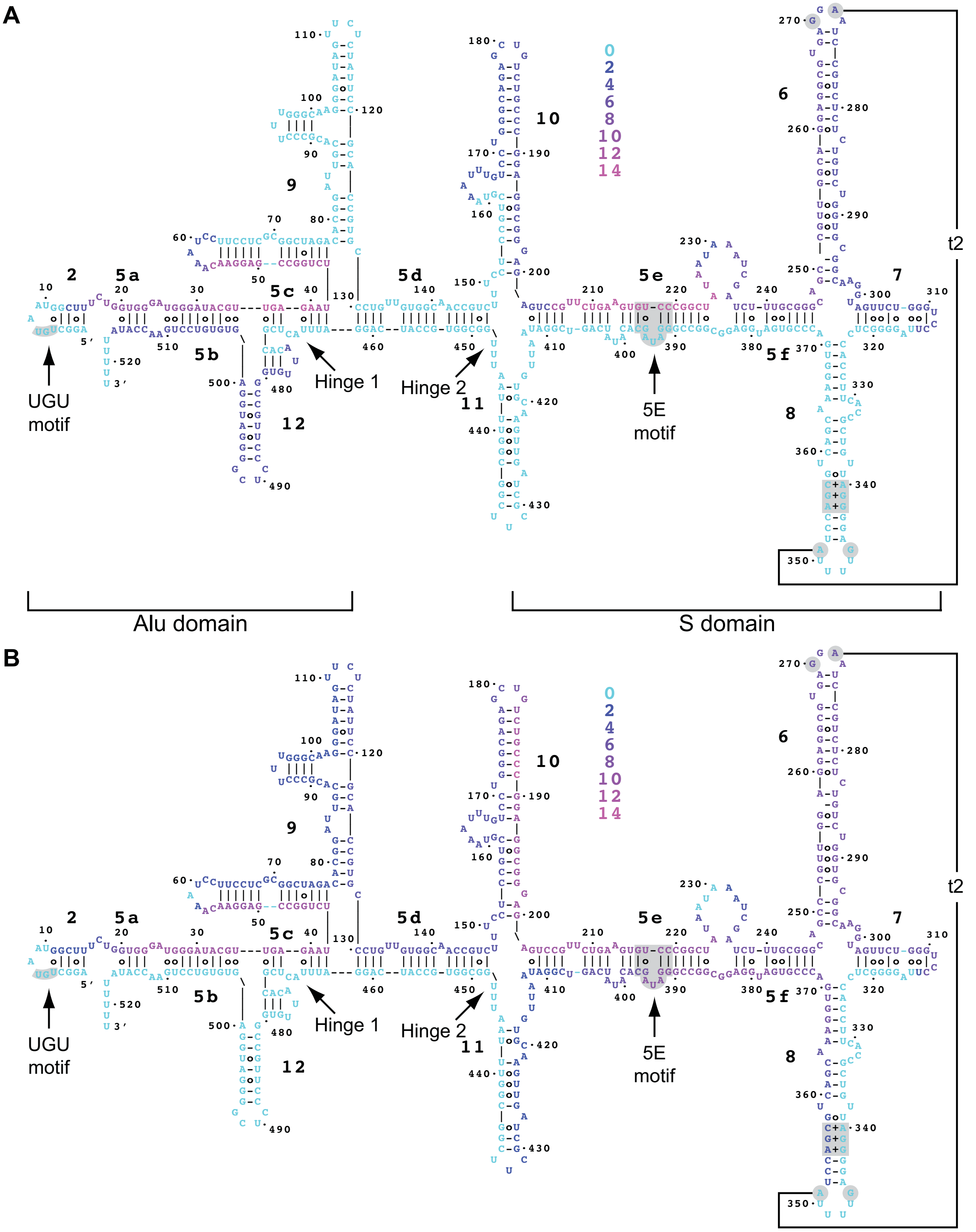

Supplementary Figure S1. Heat map of the recovered PAR-CLIP fragments.
The recovered fragments are shown on the secondary structure model of yeast SCR1 of Zwieb *et al*, where the colors indicate the frequency that the sequence was recovered [14]. The model is based on phylogenetically conserved features found in SRP RNAs and on structural probing experiments [14,15]. Yeast and other fungal SRP RNAs are unusual in that they are much larger than in other organisms, and they lack the characteristic structure consisting of hairpins 3 and 4 of the Alu domain. Yeast has the additional hairpins 9, 10, 11 and 12 that are poorly characterized and that have other proposed secondary structures. Conserved sequence

motifs and tertiary interactions are shown in gray. The colors in the legend correspond to the number of times the sequence was recovered. Ded1 was crosslinked to RNA in the presence (A) or absence (B) of glucose.

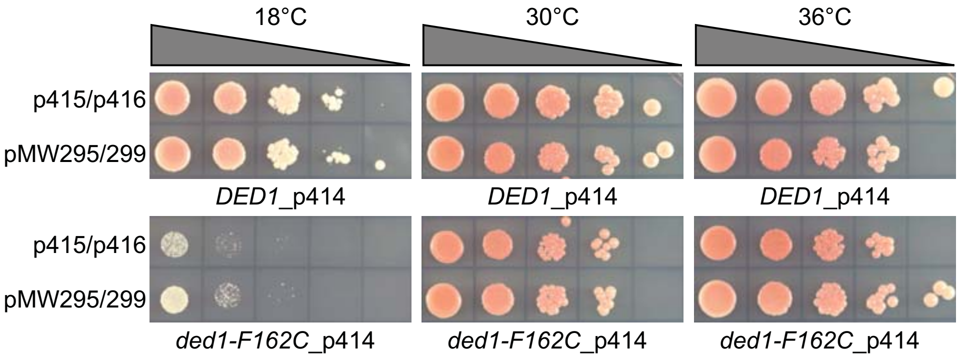

Supplementary Figure S2. Multiple-copy suppression of the ded1-F162C cold-sensitive phenotype.
The *ded1::HIS3* deletion strain containing the DED1 URA plasmid were transformed with plasmids expressing Ded1 wildtype or Ded1-F162C mutant proteins. They were subsequently transformed with pMW295 and pMW299 plasmids expressing the SRP components or with the empty plasmids. Liquid cultures were then serial diluted by a factor of 10 and spotted on synthetic defined (SD) medium plates containing 5-fluoorotic acid (5-FOA) and incubated for 3 days at 30°C and 36°C and for 5 days at 18°C

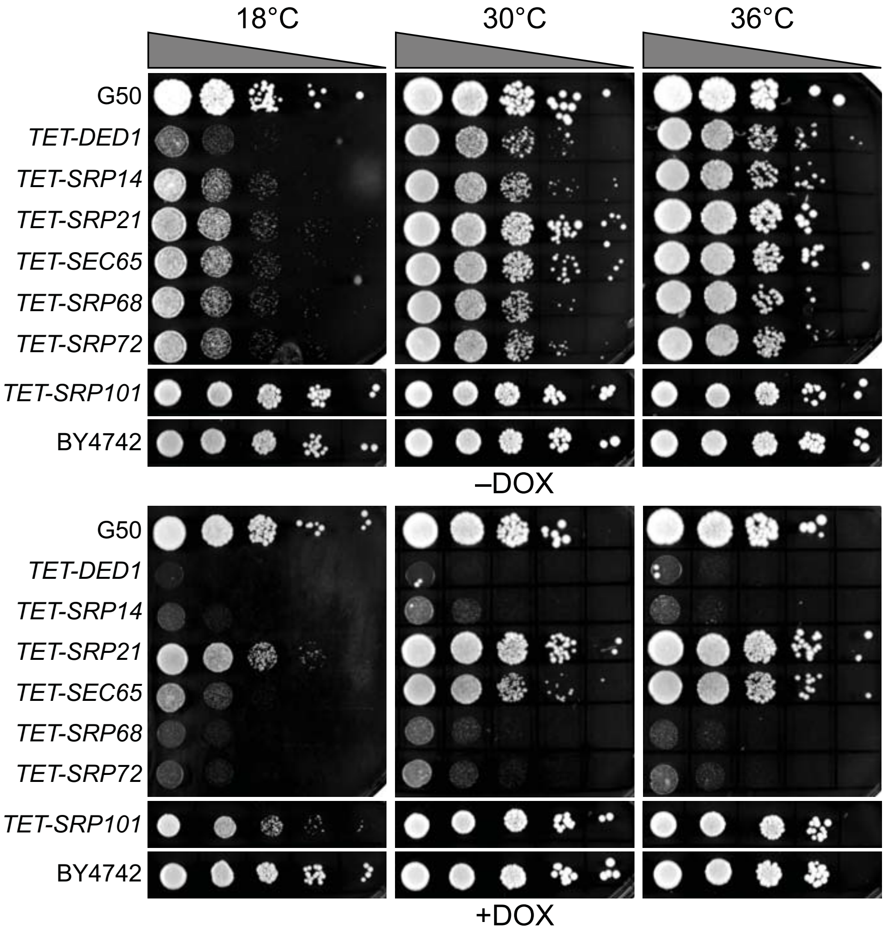

Supplementary Figure S3. Phenotypes of proteins expressed with tetracycline promoters.
Liquid cultures of the indicated strains were serially diluted by a factor of 10 and plated on YPD (yeast extract, peptone, dextrose) rich-medium agar plates, except for *TET-SRP101* and BY4742 that were plated on SD medium agar plates, in the presence (+DOX) or absence (-DOX) of 10 µg/ml of doxycycline. The G50 and BY4742 strains show wildtype growth. Plates were incubated for 2 days at 30°C and 36°C, and for 4 days at 18°C for the yeast-extract-peptone-dextrose (YPD) plates, and for 4 days and 7 days, respectively, for the SD plates.

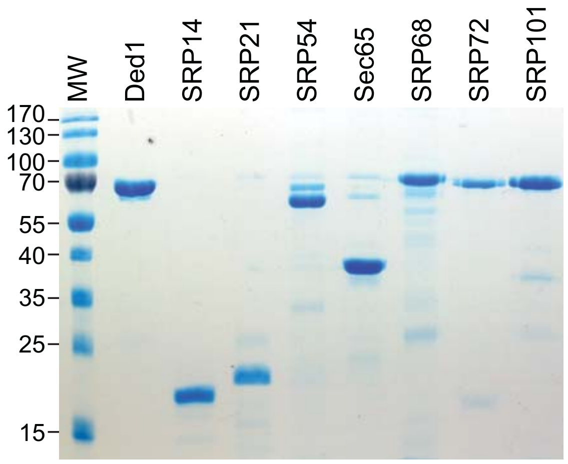

Supplementary Figure S4. Purified His-tagged recombinant proteins.
Aliquots of 1.3 µg of proteins purified on a Ni-NTA column were electrophoretically separated on a 12% PAGE and stained with Coomassie blue.

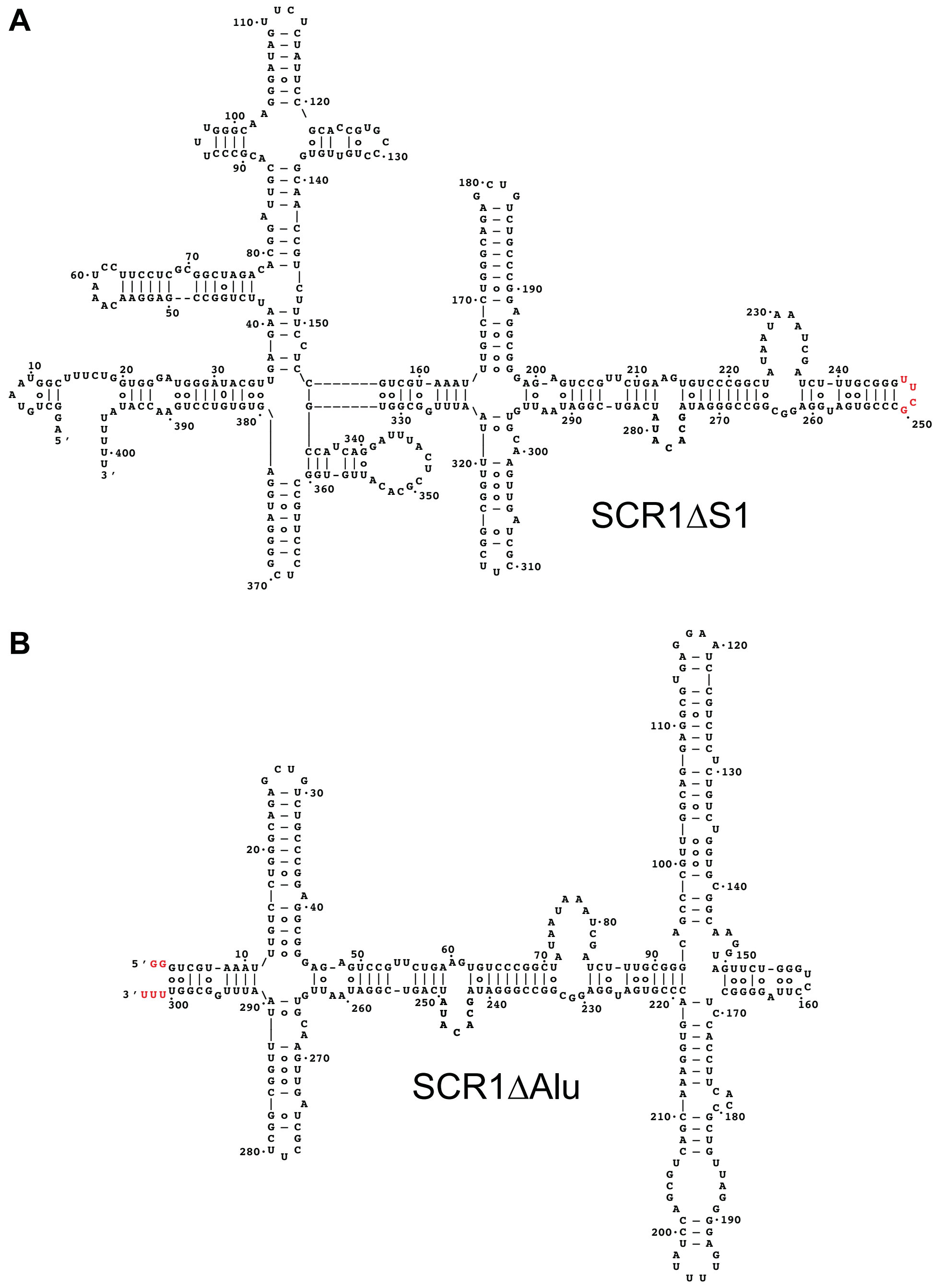

Supplementary Figure S5. SCR1 deletion constructs.
The constructs were made as described in Material and Methods of the main text. The residues shown in red were added to facilitate the construction and expression.

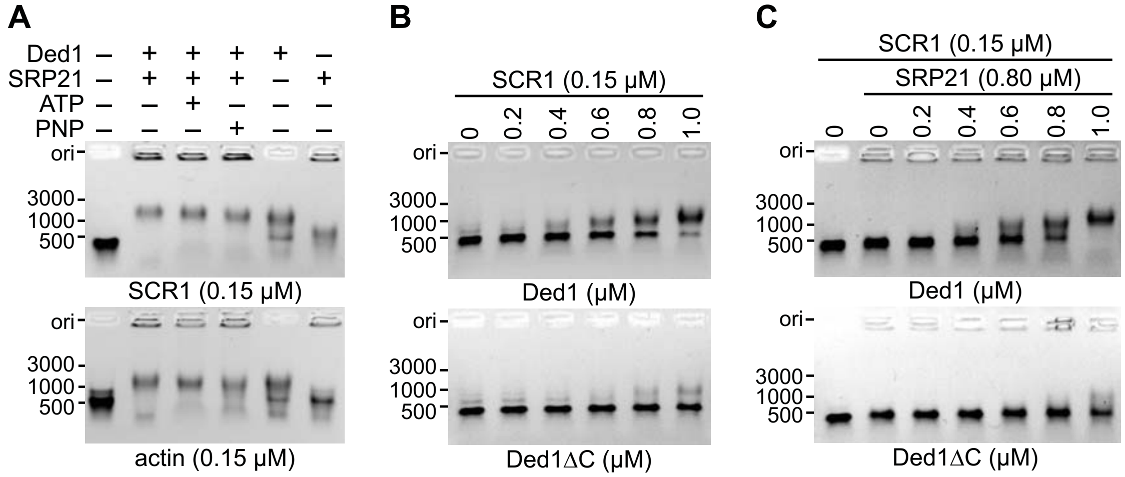

Supplementary Figure S6. Ded1 and SRP21 bound SCR1 and actin separately. Electrophoretic mobility shift assays of Ded1 with SCR1 (522 nts) and actin (605 nts) RNAs. Proteins were incubated with the RNA and separated under nondenaturing conditions on 1% agarose gels containing ethidium bromide. The markers indicate the positions of the major bands of the GeneRuler DNA ladder (Thermo Scientific). Ori, loading well of agarose gel. (A) Ded1 (0.8 μM) and SRP21 (0.8 μM) were incubated with 0.15 μM of either SCR1 or actin RNA in the presence or absence of 5 mM ATP or AMP-PNP (PNP). (B) Increasing concentrations (in μM) of Ded1 or an 78 amino-acid, carboxyl-terminal deletion of Ded1 [Ded1ΔC; (54)] was incubated with 0.15 μM SCR1 RNA and 5 mM AMP-PNP. (C) Increasing concentrations of Ded1 was incubated with 0.15 μM SCR1 RNA, 0.80 μM SRP21 and 5 mM AMP-PNP.

REFERENCES

1. Senissar, M., Le Saux, A., Belgareh-Touze, N., Adam, C., Banroques, J. and Tanner, N.K. The DEAD-box helicase Ded1 from yeast is an mRNP cap-associated protein that shuttles between the cytoplasm and nucleus. *Nucleic Acids Res*, **2014** *42*, 10005-10022. 10.1093/nar/gku584.
2. Christiano, R., Nagaraj, N., Frohlich, F. and Walther, T.C. Global proteome turnover analyses of the Yeasts *S. cerevisiae* and *S. pombe*. *Cell Rep*, **2014** *9*, 1959-1965. 10.1016/j.celrep.2014.10.065.
3. Willer, M., Jermy, A.J., Steel, G.J., Garside, H.J., Carter, S. and Stirling, C.J. An *in vitro* assay using overexpressed yeast SRP demonstrates that cotranslational translocation is dependent upon the J-domain of Sec63p. *Biochemistry*, **2003** *42*, 7171-7177. 10.1021/bi034395l.
4. Tanner, N.K., Cordin, O., Banroques, J., Doere, M. and Linder, P. The Q motif: a newly identified motif in DEAD box helicases may regulate ATP binding and hydrolysis. *Mol Cell*, **2003** *11*, 127-138. 10.1016/s1097-2765(03)00006-6.
5. Iost, I., Dreyfus, M. and Linder, P. Ded1p, a DEAD-box protein required for translation initiation in *Saccharomyces cerevisiae*, is an RNA helicase. *J Biol Chem*, **1999** *274*, 17677-17683. 10.1074/jbc.274.25.17677.
6. Cordin, O., Tanner, N.K., Doere, M., Linder, P. and Banroques, J. The newly discovered Q motif of DEAD-box RNA helicases regulates RNA-binding and helicase activity. *EMBO J*, **2004** *23*, 2478-2487. 10.1038/sj.emboj.7600272.

7. Janke, C., Magiera, M.M., Rathfelder, N., Taxis, C., Reber, S., Maekawa, H., Moreno-Borchart, A., Doenges, G., Schwob, E., Schiebel, E. *et al.* A versatile toolbox for PCR-based tagging of yeast genes: new fluorescent proteins, more markers and promoter substitution cassettes. *Yeast*, **2004** *21*, 947-962. 10.1002/yea.1142.
8. Banroques, J., Cordin, O., Doère, M., Linder, P. and Tanner, N.K. Analyses of the functional regions of DEAD-box RNA "helicases" with deletion and chimera constructs tested *in vivo* and *in vitro*. *J Mol Biol*, **2011** *413*, 451-472. 10.1016/j.jmb.2011.08.032.
9. de la Cruz, J., Iost, I., Kressler, D. and Linder, P. The p20 and Ded1 proteins have antagonistic roles in eIF4E-dependent translation in *Saccharomyces cerevisiae*. *Proc Natl Acad Sci USA*, **1997** *94*, 5201-5206. 10.1073/pnas.94.10.5201.
10. Malcova, I., Farkasovsky, M., Senohrabkova, L., Vasicova, P. and Hasek, J. New integrative modules for multicolor-protein labeling and live-cell imaging in *Saccharomyces cerevisiae*. *FEMS Yeast Res*, **2016** *16*. 10.1093/femsyr/fow027.
11. Lin, R.J., Newman, A.J., Cheng, S.C. and Abelson, J. Yeast mRNA splicing *in vitro*. *J Biol Chem*, **1985** *260*, 14780-14792.
12. Neville, M. and Rosbash, M. The NES-Crm1p export pathway is not a major mRNA export route in *Saccharomyces cerevisiae*. *EMBO J*, **1999** *18*, 3746-3756. 10.1093/emboj/18.13.3746.
13. Deshaies, R.J. and Schekman, R. A yeast mutant defective at an early stage in import of secretory protein precursors into the endoplasmic reticulum. *J Cell Biol*, **1987** *105*, 633-645. 10.1083/jcb.105.2.633.
14. Zwieb, C., van Nues, R.W., Rosenblad, M.A., Brown, J.D. and Samuelsson, T. A nomenclature for all signal recognition particle RNAs. *RNA*, **2005** *11*, 7-13. 10.1261/rna.7203605.
15. Van Nues, R.W. and Brown, J.D. *Saccharomyces* SRP RNA secondary structures: a conserved S-domain and extended Alu-domain. *RNA*, **2004** *10*, 75-89. 10.1261/rna.5137904.
